# Responsiveness of the Eating Disorders Quality of Life Scale (EDQLS) in a longitudinal multi-site sample

**DOI:** 10.1186/1477-7525-8-83

**Published:** 2010-08-11

**Authors:** Carol E Adair, Gisele C Marcoux, Theanna F Bischoff, Brian S Cram, Carol J Ewashen, Jorge Pinzon, Joanne L Gusella, Josie Geller, Yvette Scattolon, Patricia Fergusson, Lisa Styles, Krista E Brown

**Affiliations:** 1 Departments of Community Health Sciences and Psychiatry, Faculty of Medicine, University of Calgary, 1215 - 39 Ave, SW, Calgary, AB, T2T 2K6, Canada; 2 Alberta Health Services - Calgary Region, 10101 Southport Road SW, Calgary, AB, T2W 3N2, Canada; 3 Department of Human Development and Applied Psychology, Ontario Institute for Studies in Education, University of Toronto, 252 Bloor Street West, Toronto, ON, M5S 1V6, Canada; 4 Faculty of Nursing, University of Calgary, Professional Faculties Building, 2500 University Drive, NW, Calgary, AB, T2N 1N4, Canada; 5 Faculty of Medicine, University of British Columbia, 317-2194 Health Sciences Mall, Vancouver, BC, V6T 1Z3, Canada; 6 Faculty of Medicine, Department of Psychiatry, Dalhousie University, Halifax, NS, B3H 2E2, Canada; 7 Providence Health Care, 1081 Burrard Street, Vancouver, BC, V6Z 1Y6, Canada; 8 Capital Health Eating Disorder Clinic, Room 3005, AJ Lane Memorial Building, P.O. Box 900, Halifax, NS, B3K 9Z9, Canada; 9 University of Manitoba, Winnipeg, MB, R3T 2N2, Canada; 10 National Program Evaluation Services, Strategic Policy & Planning Directorate, Building M8 1 - South, 300 Merivale Road, Ottawa, ON, K1A 0R2, Canada; 11 Center for Cognitive Behavior Therapy, Department of Psychology, University of Hawaii at Manoa, Gartley Hall, Room 3, 2430 Campus Rd, Honolulu, HI, 96822, USA

## Abstract

**Background:**

In eating disorders (EDs), treatment outcome measurement has traditionally focused on symptom reduction rather than functioning or quality of life (QoL). The Eating Disorders Quality of Life Scale (EDQLS) was recently developed to allow for measurement of broader outcomes. We examined responsiveness of the EDQLS in a longitudinal multi-site study.

**Methods:**

The EDQLS and comparator generic QoL scales were collected in person at baseline, and 3 and 6 months from 130 participants (mean age 25.6 years; range 14-60) in 12 treatment programs in four Canadian provinces. Total score differences across the time points and responsiveness were examined using both anchor- and distribution-based methods.

**Results:**

98 (75%) and 85 (65%) responses were received at 3 and 6 months respectively. No statistically significant differences were found between the baseline sample and those lost to follow-up on any measured characteristic. Mean EDQLS total scores increased from 110 (SD = 24) to 124.5 (SD = 29) at 3 months and 129 (SD = 28) at 6 months, and the difference by time was tested using a general linear model (GLM) to account for repeated measurement (p < .001). Responsiveness was good overall (Cohen's d = .61 and .80), and confirmed using anchor methods across 5 levels of self-reported improvement in health status (p < .001). Effect sizes across time were moderate or large for for all age groups. Internal consistency (Chronbach's alpha=.96) held across measurement points and patterns of responsiveness held across subscales. EDQLS responsiveness exceeded that of the Quality of Life Inventory, the Short Form-12 (mental and physical subscales) and was similar to the 16-dimension quality of life scale.

**Conclusions:**

The EDQLS is responsive to change in geographically diverse and clinically heterogeneous programs over a relatively short time period in adolescents and adults. It shows promise as an outcome measure for both research and clinical practice.

## Background

Eating disorders (EDs) are serious health problems that adversely impact quality of life in adolescence and young adulthood; a critical time for individuation and establishing independence across several life domains including initiation of careers [1-3]. Unhealthy eating attitudes and dieting behaviors that elevate risk for EDs are found in nearly 30% of girls aged 10 to18 years and increases in concern with weight over time have been documented for both boys and girls aged 9 to 14 [4-6]. These trends imply that EDs will continue to be a significant health concern for the foreseeable future.

If not treated early and effectively, EDs can become chronic, and place enormous burden on the patient and his or her family [7]. Demand for treatment services is growing, along with an urgency to ground new treatments in evidence [8,9]. Treatment outcome measurement in EDs has traditionally focused on changing behavior and symptoms (e.g., reducing purging or restoring a healthy body weight) rather than on broader areas such as role functioning or quality of life, and ED experts have been calling for more holistic approaches to treatment and for broader treatment outcome measurement for more than a decade [10-12]. For example, Miller [11] characterized the traditional approach to EDs treatment outcomes measurement as "too simplistic and narrow in scope," (p. 745) and Treasure [13] wrote that "to focus merely on symptomatic relief from 'not eating', as occurs with some forms of hospital care, is primitive," (p. 212). In this paper, we describe a study to establish responsiveness in a new disease-specific quality of life (QoL) measure for EDs that taps these broader outcomes.

While broader outcomes have been measured in some research samples of ED patients using generic quality of life (QoL) instruments, including the Short-Form-36 (SF-36), the Nottingham Health Profile (NHP) and the World Health Organization Quality of Life Instrument - Brief Version (WHO QoL-Bref) [2], they have several limitations. Some domains and items on generic QoL instruments may be insensitive for some diagnoses [14], and responsiveness may be inadequate for evaluative purposes [15-17]. In addition, wording and interpretation problems with the SF-36 have been found for some patient groups including EDs [14,18,19]. QoL measurement in practice has been limited by a lack of availability of specific QoL measures for the EDs field, and as a result, many calls for a specific, relevant and responsive QoL measure have been made in the past decade [2,20-24]. In response to these identified issues, four new disease-specific instruments for EDs, including the subject of the current paper - the Eating Disorders Quality of Life Scale (EDQLS), were reported in the past three years [20,21,23,24]. An article describing an instrument to measure impairment resulting from ED psychopathology has also been recently published, but, as described, neither the important conceptual distinctions between impairment and quality of life; nor the complexity of causal pathways between symptoms and behavioural manifestation in the illness, are recognized [25]. Two of these instruments were tested in an age range that included adolescents, but adolescent-specific design methods (e.g., testing relevance of content and appropriateness of language) are reported only for the EDQLS [24].

The EDQLS was designed for an *evaluative *purpose, (i.e., to measure change over time *within *individuals) [17], such as for the assessment of patients' treatment progress and the outcomes of new treatments [11]. Given this, responsiveness is the psychometric characteristic of primary importance. According to Revicki [26], responsiveness refers to "the extent to which a measure accurately reflects change in a patient's condition," (p. 890).

Only one of the recently developed disease-specific QoL instruments for EDs has published findings on responsiveness [27]. The authors used distribution and anchor-based methods to examine responsiveness and found effect sizes around .30 (varying by subscale) for patients from three treatment programs in one city who reported improvement at one point of follow-up (12 months). These results are encouraging in suggesting that measured QoL can improve over time with treatment for EDs. However, this instrument (by Las Hayas and colleagues) emphasizes symptomatic aspects of the illness, which might be more likely to change with treatment than broader life domains [27]. It is also critical to ensure that instruments such as the EDQLS, that tap broader life domains such as leisure and relationships, are also responsive to treatment, especially when used to evaluate treatments targeted to broader outcomes. In addition, Las Hayas and colleagues did not report the use of design processes to ensure appropriateness to adolescents, so responsiveness in an instrument such as the EDQLS with this feature was warranted. The purpose of the current study was to examine responsiveness in an instrument designed to be appropriate across the full range of patient ages and which taps broad domains of QoL, across three time points for patients, including adolescents, in active treatment across multiple geographically diverse treatment programs.

## Methods

### The Longitudinal Sample

165 females and six males aged 14 years or older with a clinically confirmed diagnosis (anorexia nervosa, bulimia nervosa or eating disorders not otherwise specified) participated in the multi-site study. They came from 12 Canadian EDs treatment programs (two in Nova Scotia, three in Manitoba, five in British Columbia, and two in Alberta) providing any of inpatient, outpatient, day treatment and/or consultation to adolescent or adult patients. Approaches to treatment in these programs varied widely from inpatient medical weight restoration through individual, group or family psychotherapy based on several current therapeutic models, and supplementary therapies such as meal preparation/nutrition skill-building and recreational approaches. The intensity of current treatments and the structure of the treatment team also varied considerably. Patients were included if they had been in treatment at least two weeks and at the time of baseline measurement were at variable stages of treatment. Participants were recruited through presentations by the research assistant in group therapy sessions, and by individual clinician referrals.

### The Eating Disorders Quality of Life Scale

The EDQLS is based on the World Health Organization's definition of QoL [28] and its development was guided by published standards [26,29-33]. Content was selected to capture broad aspects of life affected by EDs and their treatment (i.e., health-related QoL), but overlap in content with instruments that measure ED symptoms and behaviors alone was avoided. Example items from the final 40-item EDQLS are *"I have a lot of rules about food" *(health related to food and weight domain (also called the eating domain) and *"I feel connected to others" *(relationships with others domain). The 12 domains or subscales are cognitive, education/vocation, family and close relationships, relationships with others, future outlook, appearance, leisure, psychological, emotional, values and beliefs, physical, and eating. Each domain has three items, except for the health related to food and weight/eating domain, which has six items plus an extra item that is similarly worded with one in the cognitive domain that was designed to be used as an internal validity check. The minimum and maximum scores are 40 and 200 respectively. The EDQLS was developed and validated for ages as young as 14, and is currently being tested in youth ages nine to 13 years. Recent work using cognitive interviewing [34-36] resulted in refinements to six items. The results reported herein relate to the first version.

A single global QoL rating: *"Please rate your overall quality of life in the last week on a scale of 1 to 10, where 1 is **Poor **and 10 is **Excellent**" *is included in a separate part of the questionnaire booklet to allow for overall construct validity assessment as recommended by Fayers and Fayers (2000) [31]. In an additional separate section of the questionnaire booklet, the 12 QoL domains are listed, and respondents are able to rate the importance of each (on a five-point scale), as well as up to two additional self-nominated domains. The importance ratings are not used to weight the total domain scores derived from the core 40 items, as per current recommendations [37], but they provide an opportunity for the patient and clinician to consider and address unique QoL issues and goals as an adjunct to the standard scores.

The total mean score on the initial validation sample (pilot and longitudinal sample at baseline - N = 171) was 110 out of a total of 200 (SD = 24.1) with higher scores indicating better QoL. Since patients were at varying stages of treatment, the baseline scores simply represent the first score for each participant. The EDQLS showed excellent internal consistency overall (Cronbach's alpha = .96) and for most subscales. Criterion validity (both convergent and divergent) was established in that sample using comparisons with the Quality of Life Inventory (Qoli) [38], Short-Form-12 (SF-12) [39], and a generic sixteen-dimensional health-related measure for youth (the 16D) [40]. Known groups validity was also demonstrated on the baseline sample, and construct validity was examined using principal components analysis and exploratory item response theory analysis. Full details on the development and initial validation of the EDQLS are available elsewhere [24].

### Validation measures and other variables

The three comparator instruments noted above - the SF-12, the QoLI and the 16D - were used to assess responsiveness across instruments for the longitudinal sample. The SF-12 is a brief version of the SF-36, an extensively tested and validated health status instrument used in many patient populations to measure health-related functioning and frequently used as an indicator of QoL [39]. Its 12 items address activities such as playing golf and climbing stairs, as well as limitations in performing physical tasks, and in working or socializing due to physical and emotional problems or pain. This measure also provides summary scores for both mental and physical health status [39]. The QoLI is a generic QoL life instrument [38]. It has 32 items that address 16 areas of life (health, self-esteem, goals and values, money, work, play, learning, creativity, helping, love, friends, children, relatives, home, neighborhood and community), and both importance and satisfaction ratings for each. It has been validated in several clinical and non-clinical populations and has good internal consistency (values ranging from .77 to .89) [38]. The 16D is also a generic QoL measure. However, it is designed specifically for youth aged 12 to 15 [40]. It covers 16 dimensions (mobility, vision, hearing, breathing, sleeping, eating, elimination, speech, mental function, discomfort and symptoms, school and hobbies, friends, physical appearance, depression, distress and vitality) with a single item for each dimension. It has good test-retest reliability and known group validity [40]. The 16D was selected for the current study to assess the appropriateness of the EDQLS in a sample that included a large proportion of adolescents (approximately one-third were under age 18 and approximately three-quarters were under age 29 at baseline). Two other standardized instruments were administered at baseline to measure general psychiatric symptom severity and ED symptom severity - the Brief Symptom Inventory (BSI) [41] and the Eating Disorders Inventory 2 (EDI-2) [42]. The BSI assesses psychiatric symptoms with 53 items in nine domains including somatization, obsession-compulsion, interpersonal sensitivity, depression, anxiety, hostility, phobic anxiety, paranoid ideation and psychoticism, and provides an overall score indicative of intensity of symptoms. The EDI-2 has 64 items in eight subscales reflecting eating disorders psychopathology/symptomology: drive for thinness, bulimia, body dissatisfaction, ineffectiveness, perfectionism, interpersonal distrust, interoceptive awareness, and maturity fears. Subscale scores and a total score are available. In this study, raw scores were used as a simple continuous variable indicator of ED symptom severity, because cut-offs for clinical significance were not provided, and individual clinical comparisons were not needed.

Other variables of interest including age, gender, diagnosis, psychiatric and medical comorbidity, prior treatment, age at first symptoms, eating disorder duration, and current program treatment duration were collected from the health record at baseline using a standard, pre-tested abstraction form. At three and six month data collection points, respondents were also asked to rate their overall health status on a five-point scale: 'much worse', 'worse', 'same', 'better' or 'much better'. They also provided supplementary information on whether they had completed or withdrawn from treatment, attributed their current status to their treatment, and whether anything other than treatment had happened that impacted their current status. The original instrument battery underwent review by clinical collaborators at the sites, as well as pre-testing with eight adolescents/young adults (aged 13 to 31) to assess burden, comprehension, and completion time.

### Data collection and management

All data were collected in person at baseline with assistance as needed, and by mail three and six months later. The follow-up protocol, based on the Dillman total design method for mailed surveys [43], included reminder letters at one week from the initial mailing, and a full study package re-mailed at three weeks, followed by phone calls to non-respondents. A final written appeal was sent to non-respondents approximately 8 to 10 weeks later. Study data were entered to an SPSS database. Error rates were measured on a 10% random sample, and confirmed to be less than 1% (mean .58% across time points). Missing data were minimal, and handled using standard decision-rules (e.g., inserting subscale means) and dual-rater agreement on items requiring judgment (such as response corrections).

### Analysis

There is currently no agreement on the optimal approach to responsiveness analysis [15,44-48]. Therefore, we calculated several indices of responsiveness and used both distribution- and anchor-based approaches. First, line and boxplots of EDQLS individual, mean total scores and subscale scores were inspected across time points. Sample differences were tested using Student's t-tests for mean differences, Pearson's chi-squared tests and (for diagnosis due to small cell frequencies) Fisher's exact test. Responsiveness was examined first using distribution-based approaches and calculated as Cohen's d, total score change, percent change and the standardized reponse mean across time periods. Next, mean score differences by time period were tested for statistical significance using a general linear model (GLM) that accounts for repeated measurement for participants with data across all time points; no other variables were included in this model because of the relatively small sample size. Responsiveness was also examined using an anchor-based approach, in which the magnitude change in total scores from baseline to the three-month time point was examined across five levels of self-reported change using a one-way ANOVA. Finally, effect sizes and standardized response means (based on absolute score changes) were calculated across time points for the EDQLS total score, for subscale scores, by age group, and for scores on the three comparator instruments. All analyses were based on the entire sample (versus comparision to a treatment as usual or untreated sample) because all participants were in active treatment at enrolment. The study was reviewed and approved by the Conjoint Health Research Ethics Board at the University of Calgary, and the respective committees for each jurisdiction.

## Results

### Sample Description

The initial 41 participants were a pilot sample for which consent had not been collected for follow-up; thus, 130 participants formed the longitudinal sample. 98 (75%) and 85 (65%) responses were received at three and six months respectively. Table 1 details patient characteristics for the baseline, three and six month samples, and the 45 participants lost to follow-up at six months. No differences were found on age, gender, diagnosis, eating disorders or psychiatric symptom severity, comorbidity, age at first symptoms, illness duration, previous treatment or time in treatment between the initial sample and those lost to follow-up at 6 months, although there may have been insufficient power for the detection of differences of the magnitude seen here, especially for variables with many categories. For example, the sample of those lost at six months seemed to include more participants with a diagnosis of bulimia and more of those who had had previous treatment. BSI and EDI-2 severity scores also appeared to be higher among those lost, yet smaller proportions had documented psychiatric and medical comorbidities.

**Table 1:** Sample characteristics: Baseline, 6 months, and for those Lost to Follow-up at 6 months

Patient Characteristic (as measured at Baseline)	All Participants (N = 130)	Those seen at 6 Months (N = 85)	Those lost to follow-up at 6 months (N = 45)
Mean Age (SD)	25.6 (10.5)	25.4 (10.3)	26.0 (11.2)^~^
Gender (n; % female)	124 (95.4)	81 (95.3)	43 (95.6)^#^
Diagnosis (n; %)			
Anorexia Nervosa - Restricting	36 (27.7)	25 (29.4)	11 (24.4)^
Anorexia Nervosa - Binge/Purge	20 (15.4)	17 (20.0)	3 (6.7)
Bulimia Nervosa	39 (30.0)	19 (22.4)	20 (44.4)
EDNOS	35 (26.9)	24 (28.2)	11 (24.4)
BSI^1 ^Global Severity Score	1.56 (.78)	1.49 (.77)	1.71 (.78)^~^
EDI II^2 ^Total Score	100.5 (45.2)	97.8 (42.8)	105.6 (49.4)^~^
Psychiatric Comorbidity (n; %)	88 (67.7)	63 (74.1)	25 (55.6)^#^
Medical Comorbidity (n; %)	45 (34.6)	32 (37.6)	13 (28.9)^#^
Age Symptoms First Appeared (years; SD)	15.3 (4.7)	15.3 (4.8)	15.3 (4.5)^~^
Previous Treatment (n;%)	86 (66.2)	54 (63.5)	32 (71.1)^#^
Mean Time in Treatment (months; SD)	12.5 (15.8)	12.9 (16.5)	11.7 (14.5)^~^
Eating Disorder Duration (years)	9.7 (9.1)	9.7 (8.8)	9.8 (9.7)^~^

^1 ^Brief Symptom Inventory

^2 ^Eating Disorder Inventory II Total Score (all subscales, clinical scoring)

~ Difference between All participants and those Lost at 6 months not significant using Student's t-tests at alpha level p = .05

# Difference between All participants and those Lost at 6 months not significant using Pearson's chi-squared test at alpha level p = .05

^ Difference between All participants and those Lost at 6 months not significant using Fisher's Exact test at alpha level p=.05

The sample included participants at a full range of stages of treatment. At baseline, 14 (17%) had been in treatment for two months or less, 28 (34.1%) for two to six months; six (7.3%) for six to 12 months; 12 (14.6%) for seven to 12 months; 10 (12.2%) for 13 to 24 months and 12 (14.6%) for longer than 24 months (one missing). Treatment status at the six-month point was reported by 76 respondents. Among those, 30 (39%) reported still being active in the same program, six (8%) active in another program, 16 (21%) had been discharged from the original program and were being followed by a family physician/GP,15 (19.7%) reported having completed all treatment, and nine (12%) withdrew. The majority of those who withdrew left for lifestyle reasons (e.g., moved or got a full-time job); only three (4%) reported that they were not benefiting from services or were otherwise unhappy with services. Overall, 67 (88%) responded positively when asked whether treatment for the ED had made their health better.

### Responsiveness According to Distribution-Based Approaches

**Figure 1:**
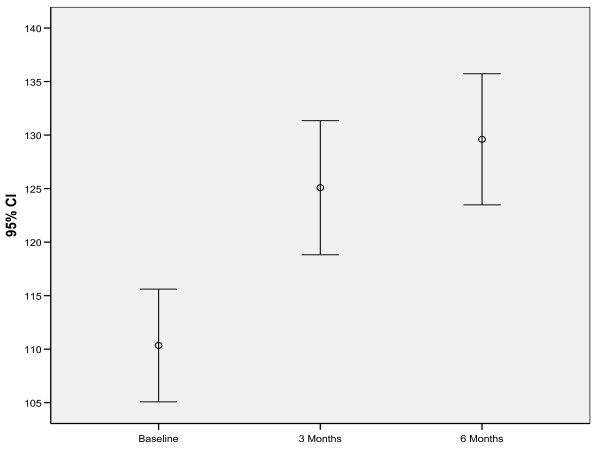
**EDQLS Total Scores at Baseline, 3 and 6 months**.

Total mean scores on the EDQLS increased from 110 (SD = 24) to 124.5 (SD = 29) at three months and 129 (SD = 28) at six months. These score differences were statistically significant (p < .001) using GLM to account for repeated measurement (Figure 1). Even though, on average, QoL scores increased, the patterns of change were highly individual. The largest increase was seen from baseline to three months, with a smaller gain from three to six months. Internal consistency of the total score was the same at all time points (Chronbach's alpha = .96). Correlations between two items in the scale tapping an identical concept but worded slightly differently and designed to indicate internal validity were also strong across time points (Pearson's r = .78, .81, and .75 respectively).

These patterns of distribution-based responsiveness held across all subscales, as shown in Table 2 with the minimum effect size for the future outlook subscale at +.44 and the maximum for education/vocation at +.89. Patterns of responsiveness, shown in Table 3, varied by age group, but effect sizes were still moderate or high for all age groups, including the youngest age group (14 to 16 years) (see Table 3).

**Table 2:** EDQLS subscale scores at baseline, 3 and 6 months and effect sizes

EDQLS Subscales	Baseline (mean SD)	3 months (mean SD)	6 months (mean SD)	Effect Size^a ^ BL to 6 months
Cognitive	8.7 (2.7)	10.1 (2.6)	10.5 (2.6)	+.67
Educational/Vocational	7.4 (2.8)	9.2 (3.1)	9.9 (3.0)	+.89
Family & Close Relationships	10.6 (2.0)	11.1 (2.3)	11.7 (2.0)	+.85
Relationships with Others	8.0 (2.4)	9.2 (2.9)	9.5 (2.8)	+.63
Future Outlook	10.3 (2.5)	11.0 (2.6)	11.4 (2.6)	+.44
Appearance	7.2 (2.8)	8.2 (3.0)	8.7 (2.9)	+.54
Leisure	10.3 (2.3)	11.4 (2.2)	11.4 (2.2)	+.48
Psychological	7.9 (2.4)	9.0 (2.6)	9.1 (2.5)	+.50
Emotional	6.7 (2.2)	7.8 (2.5)	8.0 (2.5)	+.60
Values & Beliefs	6.9 (2.5)	8.3 (2.9)	8.6 (2.9)	+.68
Physical	8.3 (2.5)	9.6 (2.7)	9.6 (2.6)	+.52
Eating	18.3 (5.1)	20.4 (5.4)	21.3 (5.7)	+.59

^a ^Effect size (Cohen's d) = 6 month mean scores minus baseline mean scores/standard deviation of baseline scores

**Table 3:** EDQLS mean scores at baseline, 3 and 6 months and effect sizes by age group^a^

Age Group (N)	Baseline (mean SD)	3 months (mean SD)	6 months (mean SD)	Effect Size^b ^ BL to 6 months
14-16 years (11)	108.0 (19.9)	132.1 (37.0)	130.2 (33.7)	+1.11
17-18 years (12)	116.0 (25.4)	132.3 (19.0)	139.3 (24.5)	+.91
19-21 years (17)	110.6 (28.3)	125.8 (29.3)	127.5 (28.4)	+.59
22-24 years (15)	105.1 (25.7)	121.1 (31.7)	123.4 (29.9)	+.71
25 years or older (28)	111.4 (22.5)	120.9 (27.4)	129.9 (26.8)	+.81

^a ^for sample with values at all three time points N = 83

^b ^Effect size (Cohen's d) = 6 month mean scores minus baseline mean scores/standard deviation of baseline scores

Distribution-based responsiveness indices for the EDQLS total score are shown in Table 4. The total score change exceeded the recommended .5 SD for responsiveness [49] and the percent change in mean scores exceeded the 10% considered to be indicative of clinically signficant change [26]. Effect sizes were moderate from baseline to three months and large from baseline to six months. Finally, responsiveness, expressed as the standardized response mean from baseline to six months was above .8, also indicating very good responsiveness [50].

**Table 4:** Distribution-based Responsiveness Indices for the Total Score

	3 months N = 98	6 months N = 85
EDQLS Total Score (SD)	124.5 (29)	129.0 (28)
Mean Scale Score changes	14.5	19.0
Mean Percent Change	13.2	17.3
Effect Size^a^	.61	.80
Standardized response mean	1.07	1.17

^a ^Effect size (Cohen's d) = 6 month mean scores minus baseline mean scores/standard deviation of baseline scores

### Responsiveness According to Anchor-Based Approaches

**Figure 2:**
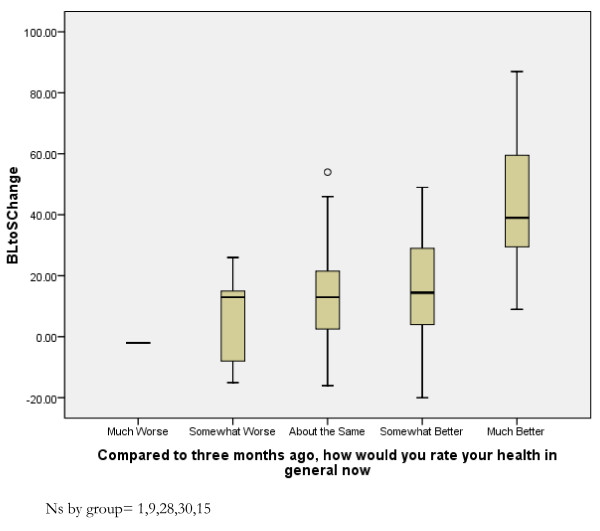
**EDQLS Change Scores, Baseline to 6 months, by Self-rated Health Improvement**.

In terms of the anchor-based approach, the magnitude of change in EDQLS total score between baseline and three months manifested an expected pattern according to five levels of self-reported change in general health between baseline and three months. Only one participant reported that their health was 'much worse', and their EDQLS total score dropped by 23 points. Those reporting that their health was 'somewhat worse' (N = 9) or 'about the same' (N = 28) had, on average, only 4.1 (SD = 17.7) and 5.4 (SD = 16.6) point increases respectively. Those reporting that their health was 'somewhat better' (N = 30) had an average 12.7 (SD = 22.4) point increase and those reporting that their health was 'much better' (N = 15) had an average 45 (SD = 22.4) point increase. The differences in mean change scores were tested using a one-way ANOVA (p < .001) (Figure 2) after re-grouping the single participant to a new category reflecting 'somewhat worse' or 'much worse' reported health status. The differences were statistically significant at the level of p < .001; Bonferroni post-hoc tests indicated that the significance level was attributable to the pair-wise comparisons of each level with the 'much better' level at at least the .05 level. To provide an indication of the amount of scale score change that corresponded to *any *reported improvement, those who rated their health as being 'somewhat better' or 'much better' by the three month point (N = 45) had an mean increase in total score from 107.6 (SD = 21.6) to 131.2 (SD = 29.2; about a 24 point improvement); whereas those who rated their health as being 'about the same', 'somewhat worse' or 'much worse' had a mean total score increase of less than five points 113.6 (SD = 26.8) to 117.9 (SD = 26.6).

### Comparative Responsiveness with Other Qol Instruments

**Figure 3:**
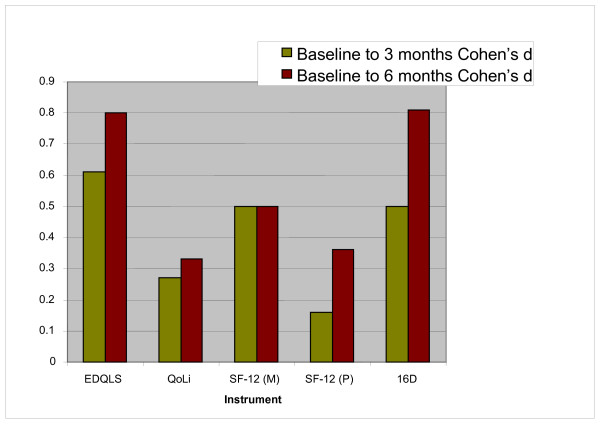
**Effect Sizes for the EDQLS and Comparator Generic QoL Instruments**. EDQLS = Eating Disorders Quality of Life Scale; QoLi = Quality of Life Inventory; SF-12 (M) = Short-Form 12 mental subscale; SF-12 (P) = Short-Form 12 physical subscale; 16D = 16 dimensional quality of life scale

Responsiveness across the follow-up period was examined graphically for the three generic QoL scales. Findings are shown in Figure 3. EDQLS responsiveness exceeded that of all comparator instruments at three months and exceeded that of all comparator instruments but the 16D at six months.

## Discussion

Our findings show that the EDQLS is responsive in a relatively short time period in a multi-site Canadian sample of EDs patients aged 14 years and older, across several indices of responsiveness. Participants were at various stages (recent admission to many months) of typical inpatient and outpatient programmatic treatment in Canada. Responsiveness was robust across subscales, and was as good or better for subscales tapping broader domains such as educational/vocational and relationship-based quality of life, as opposed to just symptoms. Patterns of responsiveness also held for both distribution- and anchor-based analyses. These findings are very encouraging, given that the sample was diverse in age and diagnosis, and was receiving a very heterogeneous range of therapies (including some inpatient care). It would be reasonable to expect the instrument to have even better responsiveness in context of a treatment trial where participants are enrolled at an early stage of treatment and the intervention is highly standardized. Establishment of responsiveness under these more ideal conditions is warranted, but, in the current study, accrual of an adequate sample size of individuals at the same stage of treatment was not feasible due to a relatively low prevalence condition and limited availability of services at this level of care.

Responsiveness has been established in a QoL scale emphasizing symptoms [27], and we have now also shown it in an instrument which emphasizes broader life domains, and for adolescents as well as adults. Effect sizes for the EDQLS were generally larger across the full sample than those reported by Las Hayas and colleagues [27] for those reporting improvement.

As expected, this disease-specific instrument outperformed the generic QoL scales for responsiveness, although, by six months, the 16D performed similarly. This suggests that the 16D may be well-suited for studies of EDs populations, where the use of a generic QoL scale is important for comparison with other patient populations and/or specialized economic evaluations. In our sample, the QoLi and the physical subscale of the SF-12 were much less responsive. During data collection, we also received spontaneous comments from participants that implied lower face validity of these tools. Thus, a responsive disease-specific instrument can now be used as an alternative or complement in research and practice. Such a measure has the additional advantage of having greater face validity and relevance in patients with ED.

Our findings are unlikely to be biased by attrition, given that the samples at each time point were very similar across a range of variables. Our response rate at our first follow-up (75%) was the same as Las Hayas and colleagues at their follow-up point [27]. Neither would there be bias due to drop-out from treatment, since we followed all participants by mail irrespective of their status in treatment. The proportion that left treatment altogether was very low over the time period studied.

Information about individuals' health status was received via self-report. Given that no corroborating data for health status ratings were collected independently, it cannot be confirmed that these were real and clinically significant changes in health status. Ideally, clinical assessments and/or BSI or EDI-2 ratings would have been taken at outcome, but this was not feasible due to large geographic distances in this multi-site study and the availability of resources to locate and visit those who were no longer in treatment programs. It is also possible that social desirability may have played a role in both score changes and self-reported improvement, if participants were motivated to please the researchers or to shed a positive light on their treatment program. However, the sample did include those who had left treatment, including for reasons of dissatisfaction with care. Finally, it is also possible that simple familiarity with the instrument may have produced the changes, although the consistency of change patterns in expected directions is reassuring.

In addition, a change in mode of collection (in person at baseline and by mail at follow-up) may have influenced results, though all collection points involved self-completion and assured confidentiality. There is evidence that collecting outcome data by mail may encourage more honest reporting, but such effects are typically small [51]. If this effect did play a role in the current study, it would have probably biased findings in the direction of lower follow-up scores resulting in less responsiveness. Even so, future research using clinical assessments of outcome and standard administration across time points is desirable.

Although, on a group basis, QoL improved significantly over the follow-up period, change trajectories in total score were highly variable, with some participants' simply maintaining gains and the QoL for some declining during the treatment period. This is consistent with a chronic disease model of EDs. Thus, caution is warranted in the interpretation of individual patient changes in scores and further work remains on establishing the minimal clinically important difference. However, the effect sizes and score ranges over time do provide some sense of the average change that might be expected in a patient population receiving publicly funded program-based ED treatment in Canada.

This study is limited by a relatively small sample size for some analyses; power was adequate for the overall analysis but was inadequate for some of the smaller differences and/or subgroup analyses. The numbers of patients with EDs, at least that seek and reach treatment, are low relative to many chronic conditions, and there is some reluctance to participate in research. These circumstances necessitated a multi-site study to accrue adequate participant numbers. This means that our results should be reasonably generalizable in terms of geography, at least in North America.

The sample included so few male patients that results cannot be considered conclusive for males. Neither can the findings be generalized to younger adolescents or diverse ethnocultural groups. Finally, the factor structure of the EDQLS has not yet been examined in an independent sample confirmatory factor analysis. Future responsiveness research on the instrument should also include larger samples and objective measurement of outcomes.

## Conclusions

The EDQLS is promising with respect to reponsiveness to change in a sample of individuals with varying diagnoses and ages; across multiple, geographically diverse treatment programs; and over a relatively short time period, and, thus, may be useful as an outcome measure for both research and practice. Further research with larger samples and using independent ratings on health status at outcome are recommended.

## List of Abbreviations

16D: The Sixteen Dimensional Health-related Measure; ANOVA: Analysis of Variance; BSI: Brief Symptom Inventory; EDI-2: Eating Disorders Inventory 2; EDNOS: Eating Disorder Not Otherwise Specified; EDQLS: Eating Disorder Quality of Life Scale; EDs: Eating Disorders; GLM: Generalized Linear Model(s); NHP: Nottingham Health Profile; QoL: Quality of Life; Qoli: Quality of Life Inventory; SF-12: Short Form-12; SF-36: Short Form-36; SPSS: Statistical Package for the Social Sciences; WHOQoL-Bref - World Health Organization Quality of Life Instrument - Brief Version

## Competing interests

The first two authors receive nominal license fees for some uses of the EDQLS.

## Authors' contributions

CA conceived and designed the study, oversaw all stages of data collection and analysis, and drafted the manuscript. GM coordinated all stages of the study, gave feedback on design, was responsible for data collection, supervised data entry, assisted with analysis and reviewed the manuscript. TB sourced literature and other background information for the manuscript and provided clinical interpretation. BC and JP provided clinical advice on design and implementation of the study, assisted with recruitment, participated in the item revision process and reviewed the manuscript. CE participated in the item revision process and reviewed the manuscript. JLG, JG, PF and YS provided clinical advice on design and implementation, research advice on validation measures, assisted with recruitment and reviewed the manuscript. LS and KEB assisted with recruitment and data collection, and reviewed the manuscript. All authors read and approved the final manuscript.
